# The dual role of empathy in clinical trial decisions

**DOI:** 10.3389/fpsyg.2025.1397581

**Published:** 2025-03-19

**Authors:** Mariam Chichua, Chiara Filipponi, Davide Mazzoni, Marco Marinucci, Marianna Masiero, Gabriella Pravettoni

**Affiliations:** ^1^Department of Oncology and Hemato-Oncology, University of Milan, Milan, Italy; ^2^Applied Research Division for Cognitive and Psychological Science, IEO European Institute of Oncology IRCCS, Milan, Italy; ^3^Department of Psychology, University of Milano-Bicocca, Milan, Italy

**Keywords:** empathy, prosocial behavior, clinical trials, decision-making, oncology

## Abstract

**Introduction:**

Empathy is a key driver of prosocial behaviors, including motivations to participate in clinical trials. Our study aimed to explore how individuals’ levels of empathy influence their intention to participate in a trial, examining scenarios where participants envision the decision for themselves (*Condition 1 - “Self”*) and when they consider a hypothetical person (*Condition 2 - "Other”*), who has to take that decision.

**Methods:**

A between-subject design was conducted on 176 healthy participants (M_age_ = 31.98, SD = 10.14). All participants responded to socio-demographic questions and were assessed for empathy. They were randomly assigned to two conditions presenting a hypothetical cancer clinical trial and assessing the intention to partake in the trial.

**Results:**

The moderation regression model was statistically significant [*R*^2^ = 0.10, *F*(7,167) = 2.04, *p* < 0.05]. Simple slopes analysis showed that for Condition 1, each unit increase in empathy was associated with 8.59 unit increase in intention to partake in a clinical trial [*b* = 8.59, SE = 4.04, 95% CI: 0.61, 16.6], whereas for Condition 2 each unit increase in empathy was associated with −9.77 unit decrease in intention [*b* = −9.77, SE = 3.78, 95% CI:−17.24, −2.3]. The slope of empathy on intention for condition 1 was significantly different than for condition 2 [Δ = 18.4, SE = 5.5, *t* (167) = 3.34, *p* < 0.001].

**Discussion:**

Empathy acts as a facilitator for partaking in trials when imagining having been diagnosed with cancer, while it becomes a barrier when thinking about another person’s participation. The reversed effect of empathy on intention shown in this study may guide future research and healthcare providers to discuss further before enrolment, involving both patients and caregivers in the decision-making process.

## 1 Introduction

Empathy has been a subject of enduring debate and exploration for many years. Empathy is defined as the ability to perceive the subjective world of the other person “as if we are” the person (Decety and Jackson, 2004; Gair, 2012; Rogers and Kinget, 2013) and could be activated by mental processes such as imagination (Decety et al., 2016). Empathy encompasses three interrelated systems crucial for caring for others: the cognitive aspect involving perspective-taking, the emotional facet characterized by empathic concern, and the motivational element associated with emotion regulation (Decety and Jackson, 2004).

According to the literature, empathy stands out as a potent catalyst for fostering prosocial behaviors—voluntary actions undertaken for the well-being of others (Penner et al., 2005). These behaviors encompass a wide spectrum, ranging from supporting friends and collaborating for the collective good to contributing to the health and public welfare (Guidi and Traversa, 2021; Grignoli et al., 2022; Penner et al., 2015).

In the context of health, the concept of empathy, often called clinical empathy (Guidi and Traversa, 2021), emerges as a crucial element. Clinical empathy implies a sense of connection with the patients, resulting from perspective-taking arising from imaginative, affective, and cognitive processes, which can also be expressed through behaviors (Tan et al., 2021). It is acknowledged for its pivotal role in comprehending the suffering of others while simultaneously emphasizing the importance of maintaining an objective distance from those experiencing distress, namely the patients (individuals who are vulnerable because of their health conditions). Neuroscientific research indicates that clinical empathy (Guidi and Traversa, 2021) necessitates at least two important conditions. First, there is the possibility for individuals to imagine themselves as if they were in the “*Others*” shoes (Thakkar et al., 2009). Second, there is the conscious awareness of the distinction between “*Self*” experiences and those of “*Others*” (Perry et al., 2013).

Cancer clinical trials stand out as an extensively researched example in the realm of prosocial motivation within clinical contexts. The primary objective of most clinical trials is the research and development of new drugs rather than catering to the specific needs of individual patients. Consequently, the motivation for engaging in clinical trials has been explored in the context of altruism, aiming to contribute to the well-being of future patients who may benefit from the developed drugs, support oncologists, and advance medical research on a broader scale (Penner et al., 2015). While some studies confirm the primary motivation to participate in clinical trials to be the will to help others (Moorcraft et al., 2016; Wegge-Larsen et al., 2023), other sources indicate that empathic feelings do not consistently drive enrollment (Catt et al., 2011; Jansen et al., 2011).

Such ambiguity in the literature guided us to adopt a different and complementary approach in investigating the impact of empathy on the decision to participate in clinical trials. Indeed, most research on the motivations for participating in a clinical trial is based on self-attributed reflections by clinical trial participants (Dainesi and Goldbaum, 2014). If this is obvious, it is impossible to exclude some bias related to subjectively reporting past experiences (while many patients invited to participate in clinical trials do not have previous experience in clinical trials).

In one of the few studies comparing participants and non-participants, the non-participants group expressed a more negative attitude toward a third person (e.g., a family member or friend) participating in a clinical trial (Madsen et al., 2002). Even if the authors did not explain clearly, we guess that this difference could be due to empathic concerns for the other’s situation. In other words, when participation in a clinical trial is related to someone else, empathy could enhance a person’s tendency to protect the other, discouraging his/her participation.

### 1.1 Aim

In this paper, we aim to clarify empathy’s complex impact on individuals’ intention to participate in a clinical trial. Specifically, we investigate how varying levels of empathy influence this intention under two experimental conditions: when individuals imagine deciding for themselves (*Condition 1 - Self*), and when they consider a hypothetical other person (*Condition 2 - Other*) making the same decision.

Based on previous literature, we hypothesize the following:

1. The interaction between individuals’ levels of empathy and the experimental condition significantly influences their intention to participate in a clinical trial. Specifically, higher levels of empathy are expected to increase intention in the Self condition but decrease it in the Other condition.
2. The intention to participate in a clinical trial does not differ significantly between the Self and Other conditions overall but is moderated by individuals’ levels of empathy.

## 2 Methods

### 2.1 Participants

*A priori* power analysis was conducted using G∗Power V. 3.1.9.2 software (Faul et al., 2009). The primary endpoint focused on the difference between the two experimental conditions in empathy and the intention to partake in clinical trials. To detect a medium effect size (*d* = 0.50), the required sample size was determined to be 176 (i.e., 88 participants in each experimental condition). The Type I error (*α*) rate was set at 0.05 (two-sided), and the Power (1 – *β*) was set at 0.90.

In this experimental study, we recruited 176 healthy adults through the Prolific platform^1^. A Qualtrics link^2^ directed participants to participate in the research. Participants were included based on the following criteria: age > 18 years, healthy general population, Italian mother tongue, ownership of a personal computer, no prior or current psychiatric or neurological conditions (self-reported during recruitment), and online approval of informed consent.

The study’s objective drove the choice of a healthy sample to investigate the psychological mechanisms of decision-making under controlled hypothetical scenarios. By focusing on healthy individuals, we aimed to isolate the role of empathy while minimizing confounding factors related to the psychological and emotional impact of a cancer diagnosis. While we acknowledge that healthy participants cannot fully replicate the lived experience of cancer patients, the hypothetical scenarios used in this study were designed to approximate decision-making in this context.

Participants voluntarily enrolled in the experiment and received modest compensation (€1.50). To mitigate potential self-selection bias, we provided participants with concise and neutral information that described the study as an investigation of decision-making processes in hypothetical scenarios without revealing the specific hypotheses or experimental conditions. This strategy aimed to avoid influencing participants’ expectations or motivations to enroll based on personal interest in the topic of clinical trials or empathy, which were central to the study. By keeping the description general, participants were less likely to self-select based on alignment with the study’s goals or assumptions about the outcomes being tested. This ensured that the sample was more representative of the general population of healthy adults targeted for this research.

The University of Milan’s ethical committee approved the study, and before participating, all participants signed the consent form to provide their informed consent.

### 2.2 Procedure

This between-subjects experimental study was conducted using the Qualtrics platform, wherein two distinct conditions were established. This experimental design was chosen to systematically manipulate the decision-making context (*Self* vs. *Other*) and examine its interaction with empathy levels. By assigning participants randomly to conditions, we aimed to ensure internal validity and minimize potential confounding factors. However, we acknowledge that the use of hypothetical scenarios might limit the external validity of our findings.

After responding to socio-demographic questions regarding sex, age, civil status, and education level and indicating their momentary distress level before the experiment, participants were randomly assigned to one of the specified conditions. Each condition presented a unique hypothetical scenario and a corresponding intention to participate in a cancer clinical trial.

*Condition 1 - Self: Scenario in first person (“Imagine yourself in the following situation. You have been diagnosed with cancer a few days ago. Followingly, as you are deciding which is the best pathway to take, your oncologist proposes to you the participation in an early phase clinical trial…”).*

*Condition 2 - Other: Scenario in third person (“Imagine that a person X is in the following situation. Person X has been diagnosed with cancer a few days ago. Subsequently, as person X is deciding which is the best pathway to take, their oncologist proposes to them the participation in an early phase clinical trial…”).*

In both experimental conditions, participants were instructed to envision either themselves (*Condition 1 - Self*) or a hypothetical individual identified as “PersonX” (*Condition 2 - Other*) grappling with a cancer diagnosis. Participants read standardized information about what clinical trials encompass and the aims of such studies, ensuring all participants had the same baseline understanding of the topic.

After the experimental phase, participants’ momentary emotional distress levels and empathy levels were assessed. Subsequently, they were asked about their intention to participate in the clinical trial under the two conditions.

At the end of the experiment, participants in Condition 2 were asked an open-ended question to specify whom they had imagined as “Person X.” This question aimed to capture the participant’s interpretation of the hypothetical individual in the scenario. Most responses indicated that participants envisioned a loved one, such as a family member, friend, or partner. These responses were not quantitatively analyzed but were used to inform the interpretation of the results discussed in the manuscript.

All data were stored respecting each participant’s anonymity. The experiment lasted 10 min to minimize participant fatigue and ensure reliable responses.

### 2.3 Measures

#### 2.3.1 Information sheet about clinical trials

An *ad hoc* information sheet was prepared, drawing upon the informed consent for clinical trials made by the National Cancer Institute - Cancer Therapy Evaluation Program (National Cancer Institute, 2018). This sheet included an overview of clinical trial’s purpose, procedures, and goals, ensuring that all participants had the same foundational knowledge before engaging with the experimental scenarios. The information sheet was not a data collection tool or scale but a preparatory material to contextualize the decision-making process.

#### 2.3.2 Empathy

Empathy was assessed using two subscales of the Italian brief version of the Interpersonal Reactivity Index (IRI) (Diotaiuti et al., 2021): Perspective-Taking Scale (PT) and Empathic Concern Scale (EC). The PT subscale measures unplanned attempts to adopt others’ points of view (5 items), while the EC subscale assesses individuals’ compassion and concern for others (4 items).

Participants were instructed to evaluate the extent to which each of the 9 statements described them, using a scale ranging from 0 (*does not describe me at all*) to 5 (*describes me completely*). The responses for each subscale were averaged to derive aggregate scores for each subdimension. To create a total empathy score, items from the Empathic Concern Scale and Perspective-Taking Scale were combined and divided by the total number of items (Tan et al., 2021). The internal consistency of the combined score was high (*α* = 0.81).

#### 2.3.3 Momentary emotional distress

The Momentary Emotional Distress scale was adapted from the Distress Thermometer scale (Grassi et al., 2013; National Comprehensive Cancer Network, 2021), a unidimensional tool for assessing distress. For this study, two items were developed based on the Distress Thermometer to measure emotional distress before and after the experiment, focusing on momentary distress rather than weekly distress. Responses were rated on a 10-point Likert scale, ranging from 0 (no distress) to 10 (maximum distress), consistent with the Distress Thermometer’s original scoring range. The mean score of the two items was used to represent the overall distress experienced throughout the experimental session. While this adaptation leverages the theoretical foundation of the Distress Thermometer, we acknowledge that the two-item scale lacks independent validation. Future research must confirm its psychometric properties and ensure its robustness in measuring momentary emotional distress.

#### 2.3.4 Intention to participate in a clinical trial

Two items were created *ad hoc* to evaluate the intention to partake in a cancer clinical trial. Responses were rated on a scale from 0 to 100.

In condition 1, the intention to participate was assessed through the following item: “*Report your intention about participating in an early phase clinical trial proposed by your oncologist?*”

In condition 2, the intention to participate was assessed through the following item: *“Thinking about Person X, report the intention that the person X may have about participation in an early phase clinical trial proposed by their oncologist.”*

### 2.4 Data analysis

Data analysis was performed using the R software (version 4.3.2). Descriptive statistics were computed for demographic variables and the main study variables. Initially, correlations between all variables of interest were calculated. Subsequently, a one-way ANOVA was run to compare the two conditions about the intention to participate in clinical trials. Then, a moderation analysis was performed to test the effect of empathy on intention between two conditions, controlling for demographic information (age, sex, education) and the level of overall momentary distress. Estimated Marginal Means (EMMs) of empathy were calculated at different levels of intention within each condition to explore the interaction between empathy and intention further. The simple slope analysis allowed for a comparison of the role of empathy in the intention between the two conditions. Socio-demographic information (age, sex, and education) and psychological variables (momentary distress) were controlled to reduce the impact of other variables that may potentially influence the intention to participate in the trial.

## 3 Results

Table 1 provides a summary of descriptive statistics for the key variables. For this study, 176 participants were recruited (M_age_ = 31.98, SD = 10.14). Sex distribution was balanced, with 86 (49%) females and 88 (50%) males. The sample demonstrated a relatively high level of education (M_edu_ = 3.99, SD = 0.90), where education was measured on an ordinal scale ranging from 1 (elementary school) to 6 (postgraduate specialization and/or PhD). Participants, on average, reported a total empathy score of 3.74 (SD = 0.74) and an intention to participate/advise in a clinical trial with a mean score of 61.06 (SD = 20.58). The overall momentary distress level was reported.

**Table 1:** Descriptive statistics.

**Variable**	**Condition 1 (Self) *N* = 88**	**Condition 2 (Other) *N* = 88**	**Total sample *N* = 176**
Age, M ± [SD]	32.42 ± [10.79]	31.54 ± [9.49]	31.98 ± [10.14]
Sex			
Male	43	45	88
Female	43	43	86
Unspecified	0	2	2
Education, M ± [SD]	4.00 ± [0.87]	3.99 ± [0.94]	3.99 ± [0.90]
Total empathy, M ± [SD]	3.87 ± [0.54]	3.70 ± [0.58]	3.79 ± [0.57]
Intention, M ± [SD]	63.16 ± [21.52]	58.93 ± [19.47]	61.06 ± [20.58]
Distress	5.58 ± [3.73]	3.81 ± [3.25]	4.70 ± [3.60]

A preliminary analysis was conducted to assess the comparability of the two groups (*Condition 1 - Self*, *Condition 2 - Other*) regarding socio-demographic characteristics (age, sex, education), baseline momentary distress, and empathy levels. No significant differences were found between the groups for these variables, confirming their comparability.

The correlation analysis reveals no significant correlations between the variables of interest (intention, total empathy, and momentary distress) in the sample. For correlations, see Table 2.

**Table 2:** Correlations between main variables and control variables.

	1	2	3	4	5	6
Age (1)	-	−0.185*	0.304**	−0.072	0.063	0.026
Sex (2)		-	−0.050	0.014	0.156*	0.001
Education (3)			-	−0.060	0.686	0.049
Distress (4)				-	0.068	0.067
Total empathy (5)					-	−0.007
Intention (6)						-

* *p* < 0.05, ** *p* < 0.01.

However, when considering the two conditions separately, significant correlations between intention and empathy were observed (condition 1: *r* = 0.21, *p* < 0.05; condition 2: −0.27, *p* < 0.05).

The one-way ANOVA tested the effect of experimental condition (*Self* vs. *Other*) on intention to participate. The analysis included the experimental condition as the independent variable and the intention to join as the dependent variable. The results showed a non-significant effect of condition on intention [*F*(1,173) = 1.86, *p* = 0.17].

As for the moderation regression analysis, controlling for socio-demographic variables (age, sex, education) and overall momentary distress, the interaction effect was statistically significant [*R*^2^ = 0.10, *F*(7,167) = 2.04, *p* < 0.05]. The interaction effect between empathy and the two conditions on participants’ intention to partake in the cancer clinical trial was significant [*b* = −18.36, *p* < 0.001]. This suggests that the impact of empathy on intention differs between the two conditions.

Table 3 shows that for Condition 1, as empathy levels increase, the mean intention to participate in the clinical trial also increases. However, for Condition 2, the mean intention to participate decreases as empathy levels increase.

**Table 3:** Estimated marginal means of empathy by condition.

**Total Empathy**	**Condition**	**Empathy EMM**	**Standard error**	**95% CI**
3.22	Self	57.4	4.31	48.9–65.9
3.79	Self	62.3	3.22	55.9–68.6
4.35	Self	67.2	3.66	60.1–74.2
3.22	Other	64.6	3.77	57.1–72.0
3.79	Other	59.0	3.10	52.9–65.1
4.35	Other	53.5	3.77	46.0–60.9

EMM, Estimated Marginal Means.

Finally, the analysis of the simple slope revealed that in Condition 1, there is a positive and significant trend between empathy and intention (*b* = 8.59, SE = 4.04, 95% CI [0.61, 16.6]), indicating that higher levels of empathy are associated with higher intentions to participate in the clinical trial. Conversely, in Condition 2, there is a negative and significant trend between empathy and intention (*b* = −9.77, SE = 3.78, 95% CI: −17.24, −2.3), suggesting that higher empathy is associated with lower intentions to recommend participation in the trial.

Empathy was measured only once after randomization. This design choice focused on participants’ empathy as a stable characteristic rather than a dynamic state influenced by the experimental task. Future studies could explore changes in empathy over time to address this limitation.

**Figure 1:**
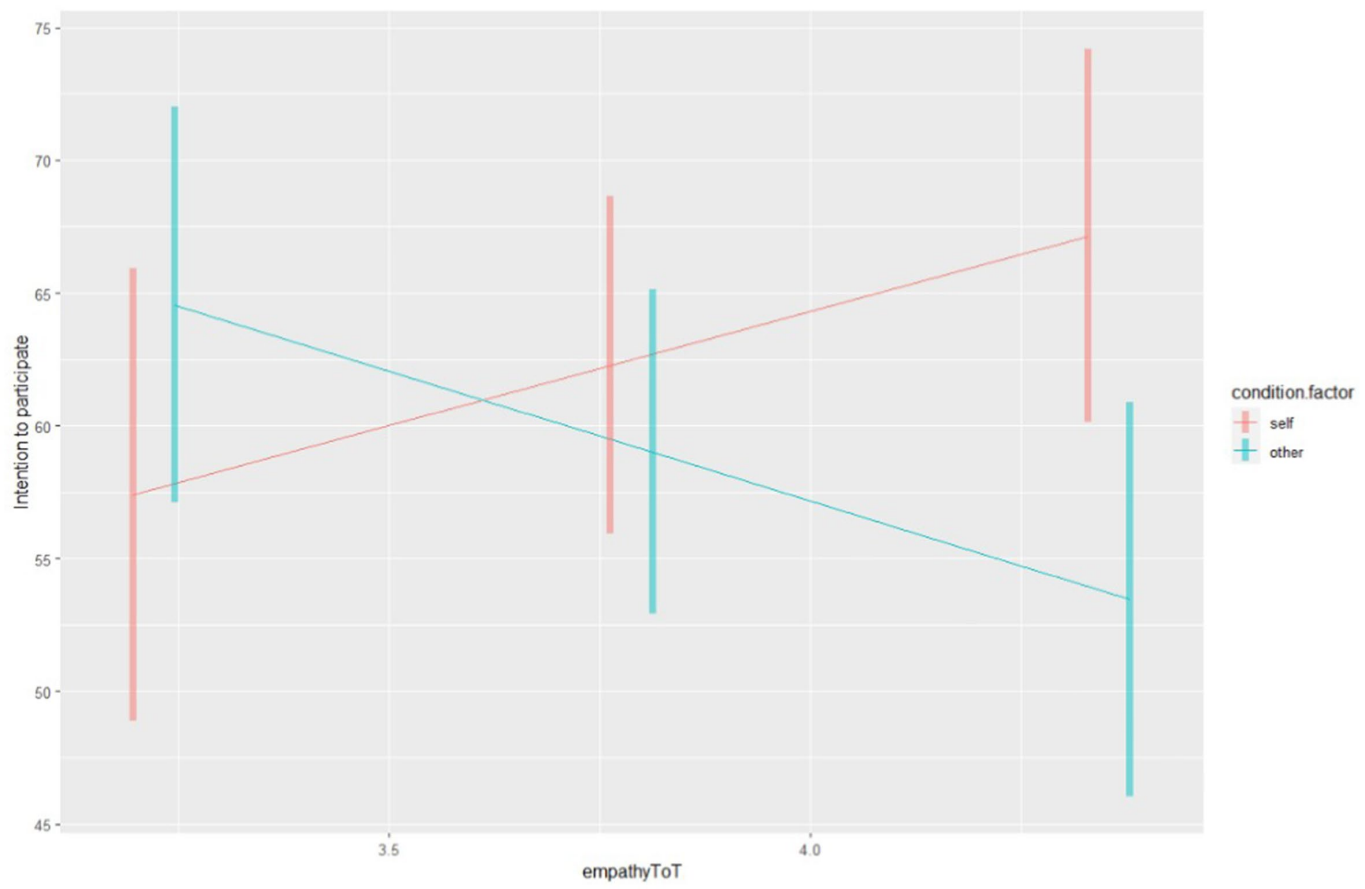
The simple slope. Empathy scores are represented on the X axis, while the intention to participate on the Y axis. The blue line corresponds to the “Other” Condition (condition 2), while the red line corresponds to the “Self” Condition (condition 1).

The contrast between the conditions indicates a significant difference in the effect of empathy on intention between Condition 1 and Condition 2 (Δ = 18.4, SE = 5.5, *t* (167) = 3.34, *p* < 0.001) (see Figure 1).

## 4 Discussion

Our study provides preliminary evidence regarding the role of empathy in shaping the intention to partake in clinical trials. While the findings highlight essential associations, they should be interpreted cautiously, considering the study’s limitations. Specifically, we observed that intention remains consistent in the two conditions. However, the direction of the effect of empathy on intention changed depending on the condition. In condition 1 (*Self*), empathy facilitated the intention to participate in a cancer clinical trial. Conversely, in condition 2 (*Other*), empathy acted as a barrier, reducing the intention of a person X to participate.

Literature has highlighted the importance of self-awareness and other awareness in the interpersonal process of empathy (Decety and Lamm, 2006), wherein emotions are shared while maintaining the ability to differentiate the origin of each emotion (Decety and Meyer, 2008). Empathy requires understanding the inner world of the person in need of care “as if” it was one’s own, a quality crucial in therapeutic contexts (Gair, 2012). These interpersonal processes were created in the two conditions of this study: one guiding the participants to focus on themselves and the other on others. This design aligns with previous research (Guidi and Traversa, 2021; Grignoli et al., 2022) that highlights the role of empathy in driving prosocial behaviors. Moreover, we made an interesting observation about how prosocial behavior translates into the two specific examples we have created for participants.

Helping other patients has frequently been named by cancer patients as one of the reasons to participate in clinical trials (Moorcraft et al., 2016; Wegge-Larsen et al., 2023; Catt et al., 2011). This may be due to the complex nature of empathy that implies self-awareness as a base for mutual connection with vulnerable individuals (Decety, 2020; Kearney et al., 2009). In other words, experiencing vulnerability may guide a person to empathize more with others experiencing the same vulnerability as well. Accordingly, in condition 1, when imagining oneself being the patient, empathy facilitated the intention to participate. The idea that one’s participation may help develop a drug from which other patients benefit is appealing to many. It gives their action a significant meaning that transcends their benefit. It makes them feel part of something bigger, part of a war against cancer (Geana et al., 2017). It is essential to consider that the risk of being overwhelmed and distressed increases in case of overidentification with another’s sufferance (Decety, 2020). Over-identification occurs when individuals deeply internalize another’s suffering, which can lead to heightened emotional distress and difficulty in maintaining an objective perspective. In line with this, on average, the distress level in condition 1 was higher than in condition 2.

On the other hand, in condition 2, the participant’s empathy acted as a barrier to intention. An explanation could be that in highly empathic participants, the concern for a hypothetical personX may have impacted how they imagined the person’s intention regarding participation. In most cases, the person that the participants imagined in our study was a loved one (e.g., family member, friend, partner, etc.). This observation brings our attention to the role of informal caregivers who share the burden of deciding on trial participation. Literature shows that informal caregivers play a significant role as supporters and advocates of their patients in trials (Castillo et al., 2021). While some caregivers encourage patients to participate (Palmer-Wackerly et al., 2018), others stop them out of concern for their health, as participation is related to numerous side effects (Brown et al., 2013). Moreover, this reluctance may act as a form of protection as participants consider the uncertainties associated with the scenario. For a caregiver, uncertainty may lead to anticipating grief and consequently increase the burden of caregiving (Li et al., 2022; Chichua et al., 2023; Filipponi et al., 2023; Filipponi et al., 2025). This underscores the complex interplay of empathy and decision-making in clinical contexts, where balancing emotional connection and rational assessment is critical for patients and their caregivers. These findings underline the need for tailored communication strategies in clinical trial settings to address empathy’s motivational and protective aspects, ensuring informed and balanced decision-making for patients and their caregivers.

Our study highlights the dual role of empathy in shaping decisions based on the target of concern. While concern for others may impact the intention to participate, studies indicate that altruism is not always a consistent driver in such decisions (Penner et al., 2015). The wish to help others is often named as a secondary reason, the first being the interest in one’s benefit from the treatment. Therefore, while acknowledging empathy’s role in such decisions, we should consider that it does not make it the driving force.

In conclusion, our study has provided insights into the role of empathy in health-related decisions, specifically in cancer clinical trials. Our findings have implications for healthcare providers who deal with the presentation of trials to their patients and their family members. When presenting the study, healthcare providers should consider the factor of empathy and how it acts differently in patients and their loved ones and, consequently, the stress associated with it in both groups. In this context, it is essential to understand the value that a multidisciplinary team can provide. Empathy for others, altruistic motivations, stress, and psychological burden are a few psychological variables among many that require the assistance of mental healthcare professionals in the oncological divisions when dealing with such delicate topics as clinical trial participation.

### 4.1 Limitations

This study has several limitations that should be addressed. First, we relied on self-report measures, which may have introduced biases such as social desirability, recall errors, and subjective estimation, potentially affecting the validity and reliability of the findings. Second, while we included an open-ended question at the end of the experiment to capture whom participants imagined as “Person X” in Condition 2, this approach was not quantitatively analyzed and relied on self-reported perceptions. A more structured exploration of this aspect could have provided a stronger empirical basis for interpreting the observed effects of empathy in Condition 2. Third, the study did not include a pilot phase to validate the hypothetical scenarios and their appropriateness before experimenting. Although the scenarios were carefully designed to approximate real-world decision-making processes, their effectiveness in eliciting intended responses remains untested. Fourth, after the experimental manipulation, empathy was measured only once as a stable characteristic rather than a dynamic state. This approach limited our ability to explore potential changes in empathy resulting from the experimental conditions. Pre-screening for baseline empathy levels and additional assessments during the experiment could have offered deeper insights into how empathy interacts with decision-making processes and allowed for greater methodological precision. Fifth, we conducted Pearson correlations between gender, coded as a binary variable (1 = male, 2 = female), and continuous variables such as empathy. While this approach is common in psychological research, it may not fully account for the categorical nature of gender, and alternative methods, such as point-biserial correlation, could have been more appropriate. Finally, while the sample of healthy individuals enabled a controlled examination of decision-making mechanisms, it does not fully replicate the lived experiences of cancer patients. Although hypothetical scenarios were used to approximate the decision-making context, future studies should consider including patient populations or caregivers to enhance the generalizability of the findings.

These limitations highlight areas for improvement in future research to strengthen the methodological rigor and validity of findings. Consequently, our results should be interpreted with caution, acknowledging these constraints.

## Data Availability

The raw data supporting the conclusions of this article will be made available by the authors, without undue reservation.

## Footnotes

^1^ https://www.prolific.co/

^2^ https://www.qualtrics.com/it/

## References

[ref1] Brown R. F. Cadet D. L. Houlihan R. H. Thomson M. D. Pratt E. C. Sullivan A.. (2013). Perceptions of participation in a phase I, II, or III clinical trial among African American patients with Cancer: what do refusers say? J. Oncol. Pract. 9, 287–293. doi: 10.1200/JOP.2013.001039, PMID: 24130251 PMC4853887

[ref2] Castillo G. Lalu M. M. Asad S. Foster M. Kekre N. Fergusson D. A.. (2021). Navigating choice in the face of uncertainty: using a theory informed qualitative approach to identifying potential patient barriers and enablers to participating in an early phase chimeric antigen receptor T (CAR-T) cell therapy trial. BMJ Open 11:e043929. doi: 10.1136/bmjopen-2020-043929, PMID: 33741670 PMC7986876

[ref3] Catt S. Langridge C. Fallowfield L. Talbot D. C. Jenkins V. (2011). Reasons given by patients for participating, or not, in phase 1 cancer trials. Eur. J. Cancer 47, 1490–1497. doi: 10.1016/j.ejca.2011.02.020, PMID: 21454072

[ref4] Chichua M. Filipponi C. Mazzoni D. Pravettoni G. (2023). The emotional side of taking part in a cancer clinical trial. PLoS One 18:e0284268. doi: 10.1371/journal.pone.0284268, PMID: 37093865 PMC10124833

[ref5] Dainesi S. M. Goldbaum M. (2014). Reasons behind the participation in biomedical research: a brief review. Rev. Bras. Epidemiol. 17, 842–851. doi: 10.1590/1809-4503201400040004, PMID: 25388485

[ref6] Decety J. (2020). Empathy in medicine: what it is, and how much we really need it. Am. J. Med. 133, 561–566. doi: 10.1016/j.amjmed.2019.12.012, PMID: 31954114

[ref7] Decety J. Bartal I. B. A. Uzefovsky F. Knafo-Noam A. (2016). Empathy as a driver of prosocial behaviour: highly conserved neurobehavioural mechanisms across species. Philos Trans R Soc Lond B Biol Sci 371:20150077. doi: 10.1098/rstb.2015.0077, PMID: 26644596 PMC4685523

[ref8] Decety J. Jackson P. L. (2004). The functional architecture of human empathy. Behav. Cogn. Neurosci. Rev. 3, 71–100. doi: 10.1177/1534582304267187, PMID: 15537986

[ref9] Decety J. Lamm C. (2006). Human empathy through the Lens of social neuroscience. Sci. World J. 6, 1146–1163. doi: 10.1100/tsw.2006.221, PMID: 16998603 PMC5917291

[ref10] Decety J. Meyer M. (2008). From emotion resonance to empathic understanding: a social developmental neuroscience account. Dev. Psychopathol. 20, 1053–1080. doi: 10.1017/S0954579408000503, PMID: 18838031

[ref11] Diotaiuti P. Valente G. Mancone S. Grambone A. Chirico A. (2021). Metric goodness and measurement invariance of the Italian brief version of interpersonal reactivity index: a study with young adults. Front. Psychol. 12:12. doi: 10.3389/fpsyg.2021.773363, PMID: 34987448 PMC8721117

[ref12] Faul F. Erdfelder E. Buchner A. Lang A. G. (2009). Statistical power analyses using G*power 3.1: tests for correlation and regression analyses. Behav Res Methods 41, 1149–1160. doi: 10.3758/BRM.41.4.1149, PMID: 19897823

[ref13] Filipponi C. Chichua M. Masiero M. Mazzoni D. Pravettoni G. (2023). Cancer pain experience through the Lens of patients and caregivers: mixed methods social media study. JMIR Cancer 9:9. doi: 10.2196/41594, PMID: 37399067 PMC10365594

[ref14] Filipponi C. Masiero M. Chichua M. Traversoni S. Pravettoni G. (2025). Navigating the emotional landscape: exploring caregivers’ journey alongside breast cancer survivors with chronic pain. Support Care Cancer 33:32. doi: 10.1007/s00520-024-09064-3, PMID: 39680180 PMC11649734

[ref15] Gair S. (2012). Feeling their stories. Qual. Health Res. 22, 134–143. doi: 10.1177/1049732311420580, PMID: 21873286

[ref16] Geana M. Erba J. Krebill H. Doolittle G. Madhusudhana S. Qasem A.. (2017). Searching for cures: inner-city and rural patients’ awareness and perceptions of cancer clinical trials. Contemp Clin Trials Commun 5, 72–79. doi: 10.1016/j.conctc.2016.12.004, PMID: 29740623 PMC5936702

[ref17] Grassi L. Johansen C. Annunziata M. A. Capovilla E. Costantini A. Gritti P.. (2013). Screening for distress in cancer patients. Cancer 119, 1714–1721. doi: 10.1002/cncr.27902, PMID: 23423789

[ref18] Grignoli N. Filipponi C. Petrocchi S. (2022). Eliciting empathetic drives to prosocial behavior during stressful events. Front. Psychol. 13, 1–8. doi: 10.3389/fpsyg.2022.963544, PMID: 36337517 PMC9632620

[ref19] Guidi C. Traversa C. (2021). Empathy in patient care: from ‘clinical empathy’ to ‘empathic concern’. Med. Health Care Philos. 24, 573–585. doi: 10.1007/s11019-021-10033-4, PMID: 34196934 PMC8557158

[ref20] Jansen L. A. Appelbaum P. S. Klein W. M. P. Weinstein N. D. Cook W. Fogel J. S.. (2011). Unrealistic optimism in early-phase oncology trials. IRB 33, 1–8. Available at: https://pmc.ncbi.nlm.nih.gov/articles/PMC3095438/, PMID: 21314034 PMC3095438

[ref21] Kearney M. K. Weininger R. B. Vachon M. L. S. Harrison R. L. Mount B. M. (2009). Self-care of physicians caring for patients at the end of life. JAMA 301:1155. doi: 10.1001/jama.2009.352, PMID: 19293416

[ref22] Li J. Sun D. Zhang X. Zhao L. Zhang Y. Wang H.. (2022). The relationship between anticipatory grief and illness uncertainty among Chinese family caregivers of patients with advanced lung cancer: a cross-sectional study. BMC Palliat. Care 21:30. doi: 10.1186/s12904-022-00925-4, PMID: 35255876 PMC8902770

[ref23] Madsen S. M. Mirza M. R. Holm S. Hilsted K. L. Kampmann K. Riis P. (2002). Attitudes towards clinical research amongst participants and nonparticipants. J. Intern. Med. 251, 156–168. doi: 10.1046/j.1365-2796.2002.00949.x, PMID: 11905591

[ref24] Moorcraft S. Y. Marriott C. Peckitt C. Cunningham D. Chau I. Starling N.. (2016). Patients’ willingness to participate in clinical trials and their views on aspects of cancer research: results of a prospective patient survey. Trials 17:17. doi: 10.1186/s13063-015-1105-3, PMID: 26745891 PMC4706669

[ref25] National Cancer Institute (2018). Cancer therapy evaluation program (CTEP). Available online at: https://ctep.cancer.gov/protocoldevelopment/informed_consent.htm.

[ref26] National Comprehensive Cancer Network. (2021). NCCN Clinical Practice Guidelines in Oncology (NCCN Guidelines) – Distress Management (Version 1.2022). National Comprehensive Cancer Network. 10.6004/jnccn.2019.0048 PMC6907687 31590149

[ref27] Palmer-Wackerly A. L. Dailey P. M. Krok-Schoen J. L. Rhodes N. D. Krieger J. L. (2018). Patient perceptions of illness identity in Cancer clinical trial decision-making. Health Commun. 33, 1045–1054. doi: 10.1080/10410236.2017.1331189, PMID: 28622019 PMC6145173

[ref28] Penner L. A. Dovidio J. F. Piliavin J. A. Schroeder D. A. (2005). Prosocial behavior: multilevel perspectives. Annu. Rev. Psychol. 56, 365–392. doi: 10.1146/annurev.psych.56.091103.070141, PMID: 15709940

[ref29] Penner L. A. Manning M. Eggly S. Albrecht T. L. (2015). “Prosocial behavior in cancer research: patient participation in cancer clinical trials” in Handbook of prosocial behavior. eds. Graziano B. Schroeder D. (Oxford, UK: Oxford University Press).

[ref30] Perry A. Shamay-Tsoory S. (2013). “Understanding emotional and cognitive empathy” in Understanding other minds: perspectives from developmental social neuroscience. eds. Baron-Cohen S. Tager-Flusberg H. Lombardo M. V. (Oxford, UK: Oxford University Press).

[ref31] Rogers C. Kinget G. M. Psicoterapia e relazioni umane. In: Cionini L. (ed) Modelli di psicoterapia, 2013, p 233. Torino, Italy: Bollati Boringhieri; (1970).

[ref32] Tan L. Le M. K. Yu C. C. Liaw S. Y. Tierney T. Ho Y. Y.. (2021). Defining clinical empathy: a grounded theory approach from the perspective of healthcare workers and patients in a multicultural setting. BMJ Open 11:e045224. doi: 10.1136/bmjopen-2020-045224, PMID: 34521657 PMC8442049

[ref33] Thakkar K. N. Brugger P. Park S. (2009). Exploring empathic space: correlates of perspective transformation ability and biases in spatial attention. PLoS One 4:e5864. doi: 10.1371/journal.pone.0005864, PMID: 19516894 PMC2688758

[ref34] Wegge-Larsen C. Mehlsen M. Jensen A. B. (2023). The motivation of breast cancer patients to participate in a national randomized control trial. Support. Care Cancer 31:468. doi: 10.1007/s00520-023-07930-0, PMID: 37452876 PMC10349774

